# The impact of single-cell RNA sequencing on understanding the functional organization of the immune system

**DOI:** 10.1093/bfgp/ely003

**Published:** 2018-03-14

**Authors:** Peter Vegh, Muzlifah Haniffa

**Affiliations:** 1 Institute of Cellular Medicine, Newcastle University, Newcastle upon Tyne, UK; 2 Department of Dermatology, Newcastle upon Tyne NHS Foundation Trust, Newcastle upon Tyne, UK

**Keywords:** RNA-seq, single-cell, monocyte, macrophage, dendritic cell, lymphocyte

## Abstract

Application of single-cell genomics technologies has revolutionized our approach to study the immune system. Unravelling the functional diversity of immune cells and their coordinated response is key to understanding immunity. Single-cell transcriptomics technologies provide high-dimensional assessment of the transcriptional states of immune cells and have been successfully applied to discover new immune cell types, reveal haematopoietic lineages, identify gene modules dictating immune responses and investigate lymphocyte antigen receptor diversity. In this review, we discuss the impact and applications of single-cell RNA sequencing technologies in immunology.

## Introduction

**Figure 1.**
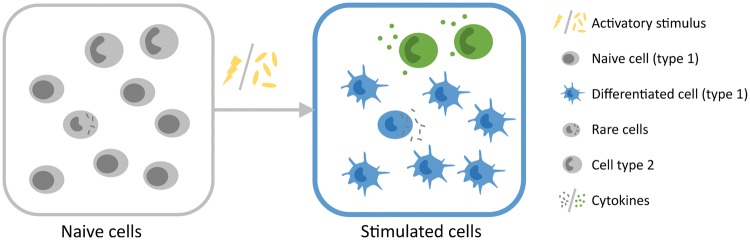
Heterogeneity of single cells within bulk populations. Cells isolated based on limited number of markers using FACS, by micropipette, optical tweezers or microfluidics, may contain multiple different cell types and cell states (left panel). If these cells are subjected to bulk analysis (squares), their heterogeneity and unique functions are masked by an average of gene expression signals. If studied individually (single cells), then their distinct activation responses to stimuli, such as cytokines, lipopolysaccharide or bacteria, can be uncovered (right panel). A specific stimulus can have variable effect on cells and leads to different outcome readout for the population in total. For instance, the rare cells may be early responders, which differentiate and activate other cells via paracrine signalling, or the cell types may respond differently, by cytokine production (cell type 2/round cells) or differentiation.

Our understanding of the immune system has been advanced over many centuries through the use of single-cell technologies, primarily microscopy and flow cytometry. However, the number of parameters that can be measured simultaneously with these methodologies is limited and reliant on a priori knowledge of which antigens to measure. The advent of next-generation sequencing to measure transcriptome at single-cell level has revolutionized our ability to interrogate the immune system [1]. This has enabled the field to move beyond the traditional cell-type classification based on limited characteristics, [2] and the averaged gene expression read out of bulk populations which may conceal biologically significant cellular heterogeneity [3, 4]. The intrinsic variation (due to cell cycle or transcription burst) and the extrinsic variation (due to exposure to stimuli or contact with other cells), and the interaction between these factors that cause heterogeneity may not be amenable to study at bulk population level (Figure 1). Accurate classification of cell types is paramount to understanding the functional configuration of the immune system.

## Single-cell genomics

Several genomics technologies such as quantitative reverse transcription PCR (RT-qPCR) and microarray have been adapted to study gene expression in individual cells [5, 6] (reviewed in [7]). While these can be cheaper, and RT-qPCR can be more sensitive for targeted sampling, scRNA-seq aims to capture the whole transcriptome and does not require preselection of target genes. Single-cell sequencing is a recent technology, but techniques to sequence genome and epigenome have been developed, such as single-cell chromatin immunoprecipitation sequencing [8], single-cell DNA adenine methyltransferase identification (DamID) [9] or single-cell combinatorial indexed Hi-C (sciHi-C), which captures chromosome conformation [10, 11]. Epigenome analysis at single-cell resolution is limited to bisulphite sequencing for methylation status (scBS-seq) and sequencing transposase-accessible open chromatin (ATAC-seq) [12, 13] (reviewed in [14, 15]). Single-cell RNA sequencing (scRNA-seq) is the most advanced method and has gained wide popularity in recent years. The general principle behind bulk and single-cell sequencing is similar with respect to mRNA extraction from single cells followed by conversion into complementary DNA libraries. However, scRNA-seq has limited starting RNA material which results in lower mapping rates and library complexity [16]. Several cell isolation methods and protocols have been developed; scRNA-seq can be performed on cells in a suspension state but also on fixed, frozen or cryo-preserved samples [17, 18]. Following tissue dissociation to generate cell suspension, individual cells can be captured and processed by droplet/microfluidics technologies (e.g. 10x Genomics Chromium, Fluidigm C1), or direct isolation into 96- or 384-well plates, by fluorescence-activated cells sorting (FACS), micromanipulation using a glass micropipette or optical tweezers [19]. Several protocols exist to generate sequencing libraries, which involves reverse transcription and polymerase chain reaction amplification of the 3′ or 5′ end or the full length of mRNA (reviewed in [20]). Utilization of unique molecular identifiers provides information on original mRNA copy number, and RNA spike-ins can be used for normalization. Plate-based approaches collect one cell in each well and importantly provide index flow cytometry information for each sorted cell. Droplet scRNA-seq approaches use beads to capture mRNA with barcoded primers. This enables high-throughput gene expression measures from high number of single cells. However, there may be cell capture bias, and mRNA-capture rate, thus library complexity, is lower than in plate-based approaches. This means fewer genes can be detected and also that gene detection saturates at approximately an order of magnitude fewer reads than in plate-based techniques [21]. Finally, because of the high number of cells sequenced and cost constraints, sequencing is often performed at lower-than-saturation depth. These factors lead to a higher gene dropout effect, i.e. non-detection of expressed genes [22]. The advantages and limitations of the different approaches present various trade-offs between the number of cells analysed, ability to capture rare cells, library complexity, depth of sequencing and cost (total and per cell). The scRNA-seq protocol of choice is therefore guided by the specific research goals (reviewed in [23]). For example, discovering distinct new rare populations requires surveying a great number of cells using droplet methods, while characterization of rare cells requires prior enriching, which can be based on known surface marker expression [24].

**Figure 2.**
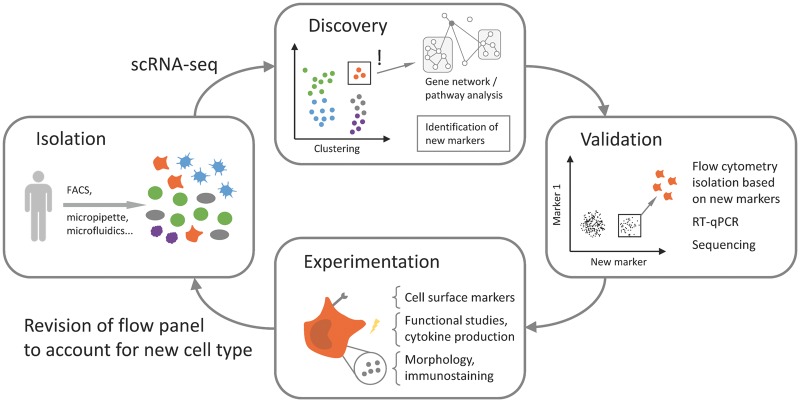
Integrated analysis of the immune system using single-cell RNA-seq. Cells from immune compartments of interest are isolated and analysed by scRNA-seq. During analysis, cells are grouped (clustered) by transcriptome profile. New and known cell clusters can be further investigated. The identity of clusters and authenticity of new cell types can be validated by flow cytometry, RT-qPCR or sequencing of isolated cells. Morphological and functional studies of new cell types or molecules provide additional biological insights. The new knowledge is integrated into subsequent experimental models.

Sequenced reads from single cells are analysed in a similar manner to bulk-population RNA sequencing data. Reads are mapped to the reference genome or transcriptome, for which a variety of programs have been developed, such as STAR, kallisto or salmon [25–27], but a reference can also be built based on the data itself. Kallisto and salmon also provide estimation of transcript abundance, but several tools, such as HTSeq [28] or featureCounts [29], provide normalized units, such as TPM, *FPKM/RPKM* or raw counts. This step is followed by data normalization, i.e. a correction of unwanted biological and technical effects, such as sequencing depth, cell cycle stage, gene number or batch effect, using spike-ins or other statistical techniques [30]. Quality checks can be performed at several stages, both at read level, where adapter sequences are trimmed and low-quality reads are removed, and at the cell level, where cells with low number of reads, genes or alignment percentage are removed [31]. Analysis of the prepared transcriptome profiles of thousands of single cells allows detailed investigations of cell diversity and heterogeneity, leading to better characterization of cell types, decomposition of tissues and even organs [32]. This heterogeneity can be explored in multiple ways. First, the data can be visualized to understand the overall structure. Single-cell RNA-seq data is multidimensional, therefore visualization requires using a dimensionality-reduction technique, such as principal component analysis (PCA), t-distributed stochastic neighbour embedding (t-SNE) [33], or a diffusion map [34]. This is followed by clustering cells according to their gene expression profiles, using data mining techniques, which include *K*-means [35], hierarchical [36], density-based or graph clustering (reviewed in [23]). It is important to minimize artefacts at the normalization stage, such as the effect of cell cycle stage, which can confound clustering analysis, and also to ensure that rare cells are clustered separately to other populations or discovered with bespoke tools such as RaceID [35] or GiniClust [37]. Finally, ‘marker’ genes can be identified for each cluster, i.e. genes that are significantly differently expressed in the cluster. These genes can be used to identify these cells for subsequent validation and functional characterization, which is performed to confirm their identity (Figure 2). Analysis of gene sets also allows to computationally characterize the cells through differential gene correlation analysis (DGCA) [38] and pathway analysis (e.g. pathway and gene set overdispersion analysis [39]), or to infer gene regulatory networks (e.g. PIDC [40], SCENIC [41]). Additionally, lineage relationships and differentiation trajectories can be reconstructed using bifurcation analysis tools, such as SCUBA [42] or Wishbone [43], diffusion maps, pseudo-time ordering of cells based on transcriptome similarity, with tools such as Wanderlust [44] or Monocle [45], or using single-cell topological data analysis (scTDA) [46]. Future approaches, such as the recently published approximate graph abstraction (AGA), will reconcile clustering and trajectory analysis to explain both discrete and continuous cell-to-cell variation [47]. Several toolkits facilitate scRNA-seq data analysis, such as Scater [48], Seurat [49] or Sincera [50]; however, the abovementioned software and statistical approaches are still currently under development and in the exploratory phase, in contrast to bulk RNA sequencing, where there is a consensus on analytical approaches.

## Immunology in the single-cell sequencing age

The key applications of scRNA-seq in immunology have been to unravel cellular heterogeneity, cell development and differentiation, haematopoiesis and gene regulatory networks to predict immune functions.

### Cellular heterogeneity

A seminal early study analysing >4000 cells from mouse spleen to dissect immune cell heterogeneity demonstrated an scRNA-seq-based classification of CD11c expressing cells [32]. scRNA-seq has since been used to study immune populations in many species, including human [51]. There are several general considerations for interpreting and validating findings from scRNA-seq analysis, in addition to specific considerations in the context of immune cells. One general consideration is resolution, i.e. number of genes per cell provided by the different scRNA-seq protocols to cluster cells by mRNA expression. Secondly, whether a transcriptionally unique cluster of cells is a distinct cell type, a transitory/intermediate cell type or cell state. Immune cells, even within distinct lineages, e.g. myeloid or lymphoid, may share expression of many gene modules, which may not necessarily be because of these cells being developmentally related. Thirdly, how to verify, isolate and functionally characterize transcriptionally distinct cell clusters. Fourth, how many individuals need to be profiled using scRNA-seq to validate cell clusters or are there alternative strategies that can be deployed to extend the findings reliably to a bigger population cohort. A recent scRNA-seq analysis using Smart-seq2 protocol of human blood lineage^-^MHCII^+^ cells consisting of known blood dendritic cells (DCs) and monocytes identified several new populations of DCs, monocytes and a DC progenitor. In this study, transcriptionally distinct cell clusters were found within what was previously thought to be a homogenous population. The identity of new cell clusters was validated by isolating cells based on surface markers predicted by mRNA expression, followed by scRNA-seq of isolated cells to validate their transcriptional identity. This enabled a cost-effective and scalable means of isolating newly identified cell types for functional characterization and demonstrating their presence as a stable cell type in a wider study population. Furthermore, by adaptively sampling peripheral blood mononuclear cells (PBMCs) using legacy knowledge of surface markers known to identify DCs and monocytes, the authors could use FACS to enrich for rare populations [52]. In contrast, scRNA-seq analysis of unselected PBMCs would require high cell numbers to be analysed to identify rare cell types because of a higher representation of abundant cell types. In the case of PBMCs, approximately 90% of profiled cells will be lymphocytes, 10% monocytes and 1% DCs. The study of 68 000 unselected PBMCs was able to identify several populations of immune cells but was more challenging for cells found at a frequency <1% [51].

In addition to exploring the overall cell census and discovering new cell types, scRNA-seq analysis can be focused on small and well-defined subsets. For example, a study of mouse Th17 cells identified heterogeneity within this population and uncovered molecular mechanisms regulating their pathogenicity [4].

### Development and differentiation

scRNA-seq has been applied to study the developmental programme undergone by haematopoietic stem cells and downstream progenitors into differentiated immune cells [53, 54] (reviewed in [55, 56]). Trajectory analysis has been applied to this continuous heterogeneity to uncover DC development and differentiation in mouse [57] and human [58–60]. Trajectory analysis can be technically challenging, as most models rely on the researcher identifying the initial progenitor/precursor population and adequate representation of cells at all stages of differentiation to define the developmental trajectory. Recent scRNA-seq-based trajectory analysis of haematopoiesis revealed a less hierarchical model of differentiation and early commitment into distinct lineages [61, 62]. In embryonic haematopoiesis, scRNA-seq revealed the early tissue colonization of precursors, which subsequently acquire a macrophage transcriptional program and differentiate into tissue-specific macrophages during organogenesis [63]. The development and heterogeneity of innate lymphoid cells (ILCs) have also been investigated [64], providing a new framework for studying these cells [65, 66]. Trajectory analysis and modelling also reconstructed the differentiation pathways of naïve CD4^+^ cells into T follicular helper (Tfh) and Th1 cells [67].

### Immune cell function

Single-cell sequencing, which provides V(D)J transcript data, has been used to study lymphocyte antigen receptors e.g. T cell receptor (TCR) and B-cell receptor (BCR) sequences. A computational method, TraCeR, was developed to reconstruct TCR sequences and reveal clonal relationships between cells. TCR reconstruction combined with T-cell transcriptional analysis was used to map T cell activation dynamics in a mouse *Salmonella* infection model [68]. Two more recent studies on TCR repertoires developed a method that can predict epitope-specificity of TCR sequences [69] and an algorithm, GLIPH (grouping of lymphocyte interactions by paratope hotspots), that groups T cells by TCR specificity [70]. Carmona *et al.* analysed evolutionary conservation of genes in human and mouse immune cell types, which enabled the identification of three T cell populations within zebrafish. Using TCR locus reconstruction, new immune-specific genes, such as novel immunoglobulin-like receptors, were discovered [71]. Similarly, a software tool, BASIC (BCR assembly from single cells), was developed for reconstructing and studying B cell repertoire [72]. Other studies focused on the lymphocyte repertoire have been reviewed elsewhere [24, 73–75].

The application of clustered regularly interspaced short palindromic repeat (CRISPR) technology-based perturbations of genes combined with scRNA-seq (Perturb-seq) has provided a new way to study transcriptional programs and gene expression networks, and was used to identify gene targets and cell states affected by individual perturbations of transcription factors in bone marrow-derived DCs in response to lipopolysaccharide [76]. Another similar combined CRISPR-based gene editing with scRNA-seq study assessed the effect of transcription factors in mouse haematopoiesis, which revealed a critical role for the *Cebpb* gene in monocyte and DC development [77]. Complex host–pathogen interactions at single-cell level have revealed new biological insights. Shalek *et al.* [78, 79] found heterogeneity in the response of bone marrow-derived DCs to the bacterial cell wall component, lipopolysaccharide, and showed bimodal gene expression across cells. Variation in host macrophage response to *Salmonella* was shown to be determined by transcriptional heterogeneity within the infecting bacteria [80, 81]. In addition, *Salmonella* growth rate was also discovered to be dependent on macrophage state [82]. Bacterial challenge of macrophages was also used in a demonstration of a new massively parallel scRNA-seq technique termed Seq-Well. In this method, cells are confined together with beads in subnanoliter wells, where cell lysis and mRNA capture to beads take place. After establishing its ability to distinguish between PBMC populations, the macrophage response to *Mycobacterium tuberculosis* was interrogated, and three macrophage sub-phenotypes were identified in the culture system [83]. A new microfluidic lab-on-a-chip method, Polaris, enabled investigation of the influence of the micromilieu on gene expression dynamics using CRISPR-edited macrophages, and implicated critical roles of SAMHD1 in tissue-resident macrophages [84].

Several other studies investigated specific aspects of immune cell function. Characterization of mouse *T*_reg_ heterogeneity uncovered their composition and identified a rare subset of CD43^+^CCR5^+^CXCR3^−^*T*_regs_ that express *Il10* and *Gzmb*, which are responsible for dampening cutaneous immune responses [85]. Ageing was shown to be associated with a diminished ability to upregulate core transcription modules for effective immune responses in naive and effector memory CD4^+^ T cells in mice [54], and a role for microbiota on epigenetic regulation and gene expression of ILCs was also recently shown by scRNA-seq analysis [86].

## Cancer immunology

In addition to infectious disease, scRNA-seq has provided new insights in cancer immunology through comprehensive tumour and immune cell profiling. Studies that combined single-cell profiling with tumour spatial heterogeneity analysis have identified multiple mechanisms of cancer-associated immunosuppression, including tumour-infiltrating T cell dysfunction [87] and immunosuppressive tumoural T cells and macrophages [88]. Similarly, a study of gene expression diversity in microglia and macrophages in glioma showed a continuum of microglia-specific versus macrophage-specific genes, and suggested that tumour microenvironments alter the gene expression profile of microglia/macrophages, so that it is dominant over cell origin [89]. Comparison of tumour with healthy tissue from the same patient offers insights into the immune landscape during tumour development. In one such study of lung adenocarcinoma, altered T, natural killer and myeloid cell compartments were identified, which possibly compromise tumour immunity [90]. Enrichment of tissue homeostatic modules in human melanoma DCs and monocytes was another mechanism of cancer immunomodulation, which was illustrated by single-cell profiling [91]. scRNA-seq analysis of 15 melanomas and additional 2068 tumour-infiltrating T cells revealed spatial and functional heterogeneity in the tumour and T cells within and between individuals, and uncovered the range of T cell activation and exhaustion programs [92]. Finally, a study involving CD4^+^ T cells found that myeloid cell expansion is critical for the control of malaria parasite (*Plasmodium chabaudi*) replication and host recovery. It established that CD4^+^ T cells produce macrophage colony-stimulating factor and that myeloid cell expansion is dependent on these cells [93].

## Complementing single-cell sequencing analysis

Single-cell RNA-seq relies primarily on a deconstructionist approach, where the analysed cells are dissociated from tissue into suspension format resulting in loss of micro-anatomical positional information. Combining scRNA-seq with spatial high-dimensional transcriptomics, imaging and single-molecule FISH (fluorescent *in situ* hybridization), such as RNA-scope, will aid dissection of functional niches and immune organisation within tissues (reviewed in [94]). The feasibility of the spatial transcriptomics approach was demonstrated on the adult mouse olfactory bulb brain region [95]. Combined strategies have been illuminating in development [96] and cancer immunology studies [90, 92]. In addition, integrating scRNA-seq with parallel lncRNA, miRNA and other omics measurements, such as epigenome, proteome or metabolome, will provide further biological and mechanistic insights [97]. Several methods have been published that measure RNA and protein from the same cells. These use oligonucleotide probes, which hybridize to target transcripts and are detected by cytometry (proximity ligation assay for RNA, PLAYR) [98], or label proteins using antibody-conjugated oligonucleotides, which are sequenced together with the transcriptome, providing a readout for a select number of target proteins (proximity extension assay, PEA [99], RNA expression and protein sequencing assay, REAP-seq [100] and cellular indexing of transcriptome and epitopes by sequencing, CITE-seq [101]). Microfluidics assays have also been developed to measure secreted proteins and transcriptome simultaneously [102].

## Future perspective

High-dimensional single-cell technologies present a radical departure from classical top-down hypothesis-based research. They enable a bottom-up unbiased approach with big data generation followed by hypothesis generation and testing. While high-dimensional single-cell methods have provided unprecedented resolution to observe and model biological phenomena, it is critical to extend the observations made with functional validation and experimentation to unravel mechanistic biological insights. This requires a multidisciplinary research team effort to overcome the convention of using genomics just as a tool, computation as an analytical means and immunology as a model system, as the combined expertise will have a greater impact than the sum of the individual components. This integrated approach will facilitate biological validation and meaningful experimentation and analysis of big scRNA-seq data sets. With time, costs associated with cell preparation and sequencing will decline and will be accompanied by progress in protocol automation and improvements in technical shortcomings of scRNA-seq, such as the small number of sequencing reads or the sparsity of data, which currently pose analytical challenges [103]. These advancements will increase cell throughput and number of genes that are detected per cell. With automation and decrease in sequencing cost, an important experiment design question to be solved is the number of biological replicates or individuals needed for reliable and generalizable conclusions.

Multi-omics approaches will provide comprehensive profiles of epigenome, transcriptome and proteome, and promise to solve some questions, such as our understanding of cell types, and the links between the genome and epigenome (reviewed in [104]). Finally, the integration of omics and screening technologies, such as Perturb-seq, will facilitate high-throughput experimentation. However, these present considerable experimental and data analysis challenges [105]. These promising approaches applied to human experimental model systems and disease settings will facilitate mechanistic rather than descriptive understanding. Such mechanistic insights can be used to identify robust molecular targets for drug and personalized immunotherapy strategies.

**Key Points**

- Single-cell sequencing technologies have revolutionized our approach to study the immune system.
- Enables high-dimensional dissection of cellular heterogeneity and establishes developmental relationships and functional predictions.
- Future integration with other types of omics data will expand our understanding of the immune system.

## Notes

**Peter Vegh** is a Research Associate at the Institute of Cellular Medicine, Newcastle University. His research focuses on the study of immune cells using cytometry and next-generation sequencing.

## Notes

**Muzlifah Haniffa** is a Wellcome Trust Senior Research Fellow in Clinical Science and Lister Research Fellow based in Newcastle University. Research in her group focuses on uncovering the functional diversity of mononuclear phagocytes in health and disease.
